# Investigation of MiR-92a as a Prognostic Indicator in Cancer Patients: a Meta-Analysis

**DOI:** 10.7150/jca.30313

**Published:** 2019-07-23

**Authors:** Yizhong Peng, Donghua Huang, Xiangcheng Qing, Lu Tang, Zengwu Shao

**Affiliations:** 1 Department of Orthopaedics, Union Hospital, Tongji Medical College, Huazhong University of Science and Technology, Wuhan 430022, China; 2 Musculoskeletal Tumor Center, Department of Orthopedics, The Second Affiliated Hospital of Zhejiang University School of Medicine, Hangzhou, P.R. China; 3 Department of Hematology, Union Hospital, Tongji Medical College, Huazhong University of Science and Technology, Wuhan 430022, China

**Keywords:** miR-92a, prognosis, clinical characteristics, meta-analysis, cancer.

## Abstract

**Background:** MiR-92a has been discovered to be involved in the malignant behavior of various types of cancers. However, the particular clinical and prognostic roles of miR-92a in tumors still need to be identified more precisely. The current meta-analysis assessed the prognostic value of miR-92a in various carcinomas.

**Methods:** Systematic literature searches of PubMed, PMC, Web of Science (WOS), Embase in English and Wanfang, SinoMed and the China National Knowledge Infrastructure (CNKI) in Chinese up to Jan 15^th^ 2019 were conducted for eligible studies. Twenty studies involving a total of 2573 patients were included in the analysis. Pooled hazard ratios (HR) for overall survival (OS) and disease-free survival (DFS), progression-free survival (PFS) and recurrence-free survival (RFS) were assessed using fixed-effects and random-effects models. Meta-regression and subgroup analyses were carried out to explore the source of heterogeneity. Odds ratio (OR) and 95%CIs were applied to evaluate the relationship between miR-92a expression levels and clinicopathological characteristics.

**Results:** A significant association between miR-92a levels and OS (HR=2.18) was identified. The random pooling model also revealed significance of consistency (HR=2.14), indicating that the stability of the results. Subgroup analyses were performed and the corresponding significance was recognized in Chinese cancer patients (HR=2.35), studies of specimen derived from tissues (HR=2.43), non-hematological cancer (HR=2.35), osteosarcoma (HR=2.54), non-small cell lung cancer (HR=2.33), hepatocellular carcinoma (HR=2.40) and so on. There were significant relations observed of the expression level of miR-92a to tumor size (≥5 vs <5 cm) (OR=2.13), lymph node metastasis (present vs. absent) (OR=1.87), distant metastasis (present vs. absent) (OR=2.99) and so on.

**Conclusions:** the over expression of miR-92a is associated with unfavorable prognosis of Chinese cancer patients. In addition, patients of elevated miR-92a expression level are likely to develop the cancers of more malignant behaviors.

## Introduction

Cancer has been a leading cause of death in both developing and developed countries recently^1^. It has reported 1,688,780 new cancer patients and 600,920 cancer deaths in the United States in 2017^2^. Although diagnostic capability and therapeutic method of cancers have been considerably developed recently, the prognosis of cancer patients, especially those in the advanced stages of tumor, is still highly unsatisfied. These could partly attribute to a lack of efficient prognostic biomarkers which could guide the clinician in the early treatment of cancer patients.

MicroRNAs (miRNAs), a kind of highly conserved non-coding RNAs, regulate target genes expression at post-transcriptional level^3^-^7^. More than half of the genes which codes miRNAs are located in cancer-related genomic regions or fragile areas. MiRNAs could function as either oncogenes or anti-oncogenes and are related to various types of human cancers^8^, ^9^. A growing evidence has revealed the involvement of miRNAs in human cancers' development and progression, including apoptosis^10^-^12^, proliferation^10^, ^13^, ^14^, the cell cycle^15^, ^16^, metastasis^17^, ^18^, etc.

A clinically relevant and efficient prognostic biomarker could denote the progression and metastasis of the underlying cancers and help clinicians to make a more appropriate treatment strategy for cancer patients. MiR-92a, one member of the miR-17/92 cluster, participates in the regulation of cell proliferation, immunity, development and tumorigenesis^19^. Recently, miR-92a has been observed to be closely related to the prognosis of cancer patients. Most studies have explored that a poor prognosis of cancer patients comes with an upregulated expression of miR-92a in tumor tissues or blood. There are a number of studies reporting that patients with a high expression level of miR-92a experience a low survival rates or quick tumor progression and metastasis in colorectal cancer^20^-^23^, non-small cell lung cancer^24^, ^25^, osteosarcoma^26^, ^27^, hepatocellular carcinoma^28^, ^29^, gastric cancer^30^-^32^, esophageal squamous cell carcinoma^33^, multiple myeloma^34^, non-muscle invasive bladder cancers^35^ and nasopharyngeal carcinoma^36^. Nevertheless, some emerging studies have identified that an increased miR-92a level was closely linked to a favorable survival: Nilsson et al.^37^ pointed out that upregulation of miR-92a was associated with decreased tumor macrophage infiltration and better outcomes in breast cancer. Papageorgiou et al.^38^ reported miR-92a overexpression as an independent predictor for better survival outcomes of patients in chronic lymphocytic leukemia. Slattery et al.^39^ observed similar results in colorectal cancer. However, Xu et al.^40^ found a lack of relationship between miR-92a and prognosis of esophageal squamous cell carcinoma. These outcomes indicated that the observed associations might be inconsistent because of different miRNAs detection methods, cut-off values, follow-up time, specimens or other possible factors.

## Materials and Methods

### Search strategy

We carried out a literature search using the online databases including PubMed, PMC, Web of Science (WOS), Embase in English and VIP, Wanfang, SinoMed and the China National Knowledge Infrastructure (CNKI) in Chinese from inception to Jan 15^th^ 2019. The following strategy was applied: (cancer [Title/Abstract] OR Tumor [Title/Abstract] OR Neoplasm [Title/Abstract] OR Neoplasia [Title/Abstract] OR Osteosarcoma [Title/Abstract]) AND (MicroRNA [Title/Abstract] OR miRNA [Title/Abstract] OR mir*[Title/Abstract]) and 92a [Title/Abstract]. The reference lists of included studies were also examined manually. Two authors (Yizhong Peng and Donghua Huang) independently screened the titles and abstracts of all retrieved records to rule out irrelevant articles. The remaining studies were evaluated by full-text scanning. Any inconsistency was resolved by discussion or consulting to a senior author (Xiangcheng Qing).

### Inclusion and excluded criteria

The inclusion criteria were: (1) studies identifying the association between miR-92a expression and human cancer prognosis and clinical features; (2) studies reporting sufficient data to calculate the hazard ratio (HR) and its corresponding 95% confidence intervals (CIs); (3) studies published in English or Chinese. (4) Retrospective, prospective or ambispective cohort studies. Studies were excluded if: (1) they were animal studies, case reports, reviews, letters, abstracts, comments and expert opinions; (2) they did not contain enough survival data or relative clinicopathological parameters; (3) they were not published in English or Chinese; (4) they were not relevant to the prognosis of human cancers.

### Data extraction

Based on the inclusion and exclusion criteria, data extraction from the enrolled studies was managed separately by two investigators (Yizhong Peng and Donghua Huang). Any disagreement was overcome by discussion or inquiry to a senior author (Xiangcheng Qing). For each eligible study, the following characteristics were collected: the first author, year of publication, country, tumor type and clinical stage, number of patients included, the type of specimen, detection methods of mi-92a expression levels, follow-up time, cut-off values, survival analysis and their source of HR, HR for overall survival (OS), disease free survival (DFS), progression-free-survival (PFS) and relapse free survival (RFS) as well as 95%CIs and the quality of study. Additionally, the clinicopathological features of including subjects were collected from the eligible studies. For those studies with only Kaplan-Meier curves available, data were extracted from the graphical survival plots based on the described approach^41^, ^42^. For studies with HR and 95%CI reported, we extracted the data of univariate (log rank tests) and/or multivariate (cox regression) separately.

### Quality assessment

All studies included in the current meta-analysis were cohort studies. The Newcastle-Ottawa scale (NOS) was applied to identify the quality of studies^43^. The score ranges from 0 to 9. A study with a score larger than 6 was regarded as methodologically high quality. Three authors (Donghua Huang, Yizhong Peng, Xiangcheng Qing) assessed qualities of recruited articles independently and accordant NOS scores were reached for each article by discussion.

### Statistical analysis

HR and 95%CIs was utilized to assess the prognostic value of miR-92a on various types of human cancers. The adjusted HRs (95%CIs) for OS, DFS, PFS and RFS were computed using data extracted from the cox regression model as well. The pooled HR > 1 and 95% CIs not overlapping 1 in the forest plot denoted that cancer patients with increased miR-92a had a poor prognosis. Heterogeneity evaluation was conducted using Cochran's Q test and Higgins's I^2^, I^2^> 50% and p-value < 0.10 indicating a significant heterogeneity.^44^ Both fixed pooling model and the random pooling model was applied in the analysis. Subgroup analyses leveled by population (Chinese and Greek), sample size (≥100 and <100), NOS scores (≥8 and <8), specimen (blood and tissues), tumor category 1 (gastrointestinal cancer and non-gastrointestinal cancer) and tumor category 2 (hematological cancer and non-hematological cancer) was implemented. Sensitivity analysis was managed by omitting each study in turn to test the stability of the results. Potential publication bias was assessed by visually evaluating the asymmetry of the funnel plot, Egger's linear regression test and Begg's funnel plot test^45^. The odds ratios (ORs) and its corresponding 95%CIs were also calculated to test the linkage between miR-92a expression and clinicopathological characteristics. All statistical analyses were conducted by Stata 14.0 (Stata Corporation, College Station, TX, USA). All two-tailed p-value < 0.05 was considered as statistically significance, except those for heterogeneity.

## Results

### Searching results and study characteristics

**Figure 1:**
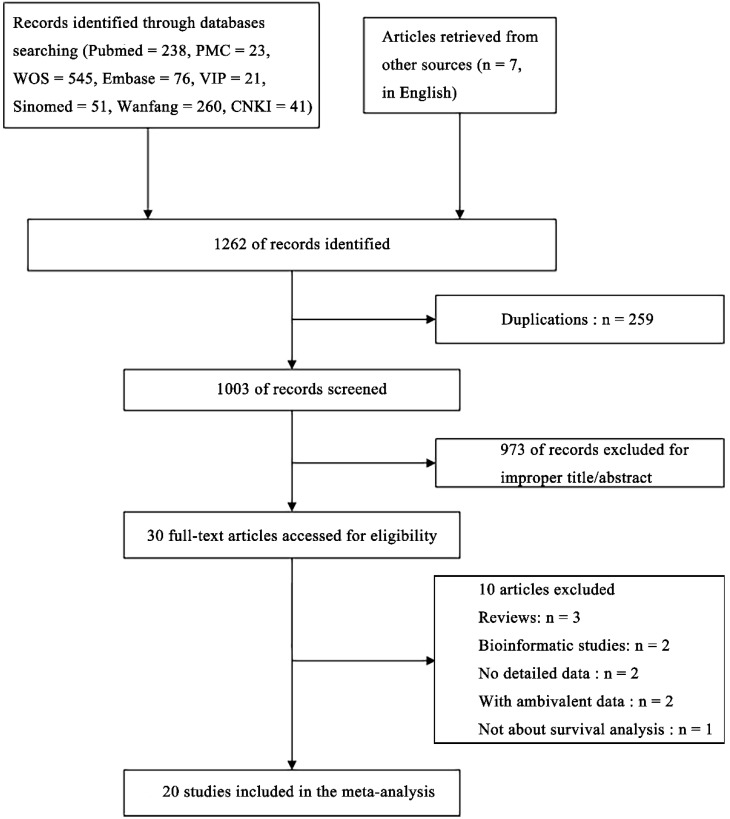
Flow chart of the meta-analysis

Twenty studies^20^-^31^, ^33^-^40^ involving a total of 2573 patients were included for the present meta-analysis (Figure 1). The characteristics of included studies were summarized in Table S1. Five studies evaluated colorectal cancer, two studies assessed esophageal squamous cell carcinoma, two studies explored osteosarcoma, two studies identified hepatocellular carcinoma, two studies focused on non-small cell lung cancer, two studies evaluated gastric cancer, and one each explored multiple myeloma, non-muscle invasive bladder cancers, breast cancer, chronic lymphocytic leukemia and nasopharyngeal carcinoma. The studies were performed in five countries (China, Spain, Sweden, Greece and USA) and published from 2010 to 2018. Thirteen studies reported available HRs and the 95% CIs, whereas the remaining seven studies only provided Kaplan-Meier curves, from which we could calculate the HRs. There were 17, 3, 2, 2 studies for OS, DFS, PFS and RFS, respectively.

### MiR-92a expression levels as an indicator for overall survival (OS)

**Figure 2:**
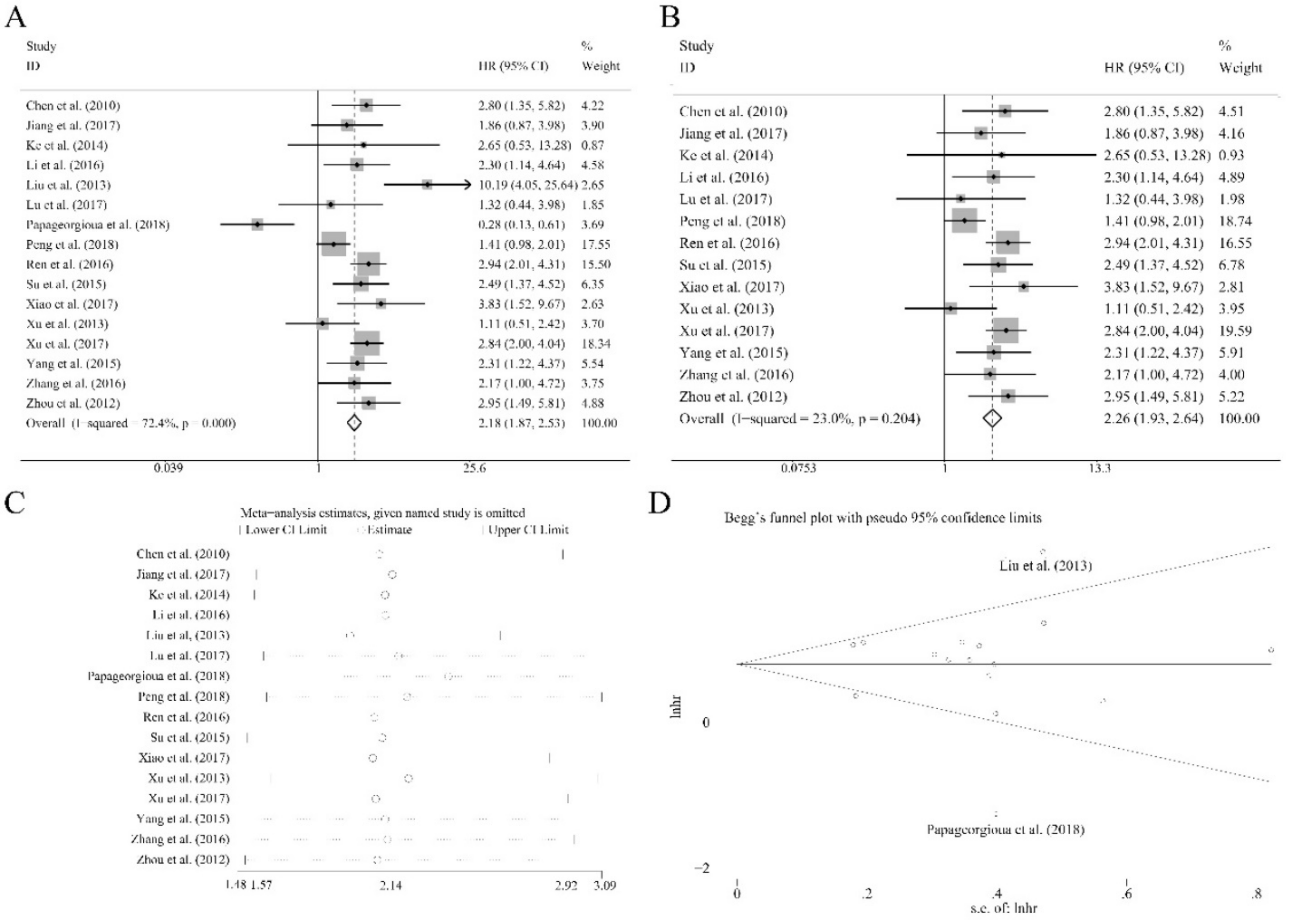
Association between miR-92a expression levels and (A) overall survival and (B) overall survival without the outliers as well as corresponding (C) sensitivity analysis and (D) publication bias evaluation

Sixteen recruited studies including 1944 patients evaluated the prognostic value of miR-92a expression levels to the outcome parameter (OS) using log rank tests and presented the data of univariate. In general, a significant association between miR-92a levels and OS (HR=2.18, CI: 1.87-2.53, Figure 2A) was identified. However, an obvious heterogeneity was also observed within the analysis (I^2^=72.40%, P<0.10, Table 1). Next, the random pooling model was implemented in succession and the significance was still consistent (Table 1), indicating that the stability of the results. Next, the sensitivity analysis was conducted, and there was no study that had significant impacts on the results (Figure 2C). In addition, funnel plots, Begg's rank correlation and Egger's weighted regression method were implemented to evaluate the publication bias. We identified two researchers as the outliers (Figure 2D), which were Liu et al.^21^ and Papageorgioua et al.^38^ The removal of the outliers greatly reduced the heterogeneity in the overall analysis and the significance of the prognostic effects of miR-92a was still obvious (Figure 2B).

**Figure 3:**
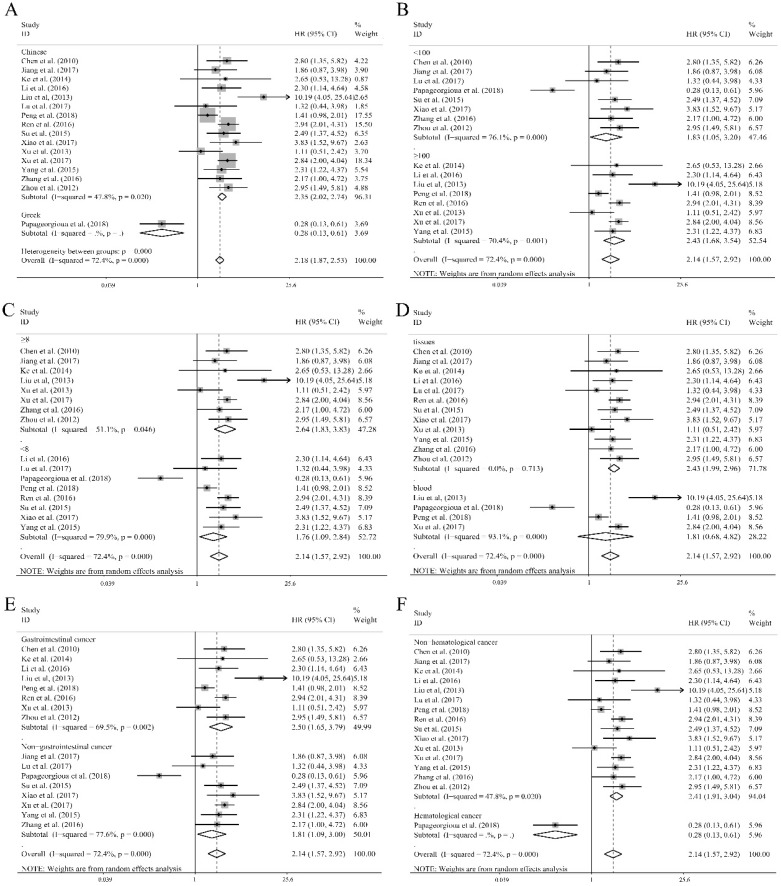
** Subgroup analyses of (A)** population (Chinese and Greek), **(B)** sample sizes (<100 and ≥100), **(C)** NOS scores (<8 and ≥8), **(D)** specimen (tissues and blood), **(E)** tumor category (gastrointestinal cancer and non-gastrointestinal cancer), **(F)** tumor category (hematological cancer and non-hematological cancer) for overall survival

To further demonstrate the source of heterogeneity, subgroup analyses was applied, and the heterogeneity was diminished within the studies of Chinese population (I^2^=47.80%, P=0.020, Table 1) and the corresponding relation of miR-92a levels to OS was significant (Figure 3A). Moreover, the homogeneity was achieved in the studies of specimen derived from tissues (I^2^=0.00%, P=0.713, Table 1) and the corresponding significance was recognized (Figure 3D). Also, the non-hematological cancer, osteosarcoma, non-small cell lung cancer and hepatocellular carcinoma group revealed eliminated heterogeneity as well (I^2^=47.8%, P=0.020; I^2^=28.40%, P=0.237; I^2^=40.80%, P=0.194; I^2^=0.00%, P=0.866, respectively, Table 1), and the significant association was also obvious in non-hematological cancer (Figure 3F), osteosarcoma (Figure S1), non-small cell lung cancer (Figure S1), hepatocellular carcinoma (Figure S1). In addition, significant associations were observed between miR-92a expression levels and OS in the studies with sample size less than 100 or greater than or equal to 100 (Figure 3B), NOS scores less than 8 or greater than or equal to 8 (Figure 3C), gastrointestinal cancer (Figure 3E) or non-gastrointestinal cancer (Figure 3E) by random pooling model, which were consistent to the significance of the results by fixed pooling model (Table 1). MiR-92a expression level was found to be related to the prognosis in the patients of all the cancers listed in Table 1, when fixed pooling model was implemented, and the results were stable with random pooling model except for the gastric cancer and esophageal squamous cell carcinoma (Figure S1).

Meta regressions were further implemented to explore the source of heterogeneity. However, no subgroup factors had posed significant impacts on the variation of HRs (Table 1).

### The independent role of miR-92a expression level as a prognostic indicator

**Figure 4:**
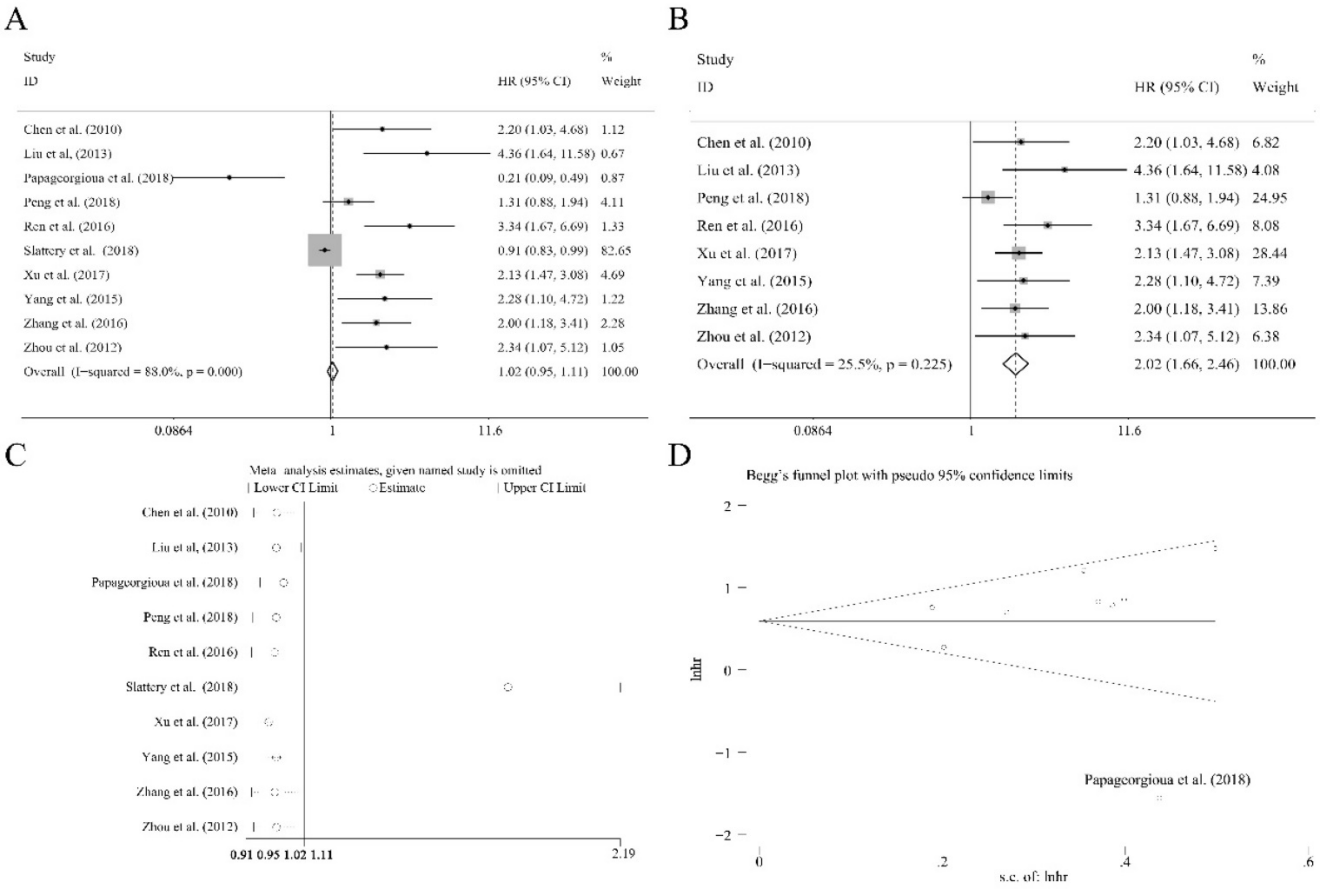
** The independent role of miR-92a as a prognostic indicator for (A)** overall survival, **(B)** overall survival without outliers, **and (C)** sensitivity analysis, **(D)** publication bias evaluation

Ten researches containing 1519 patients utilized the cox multivariate regression to evaluate the independent prognostic value of miR-92a expression levels in cancer patients by adjusting other factors. There was no significant relation of miR-92a expression level to the OS (Figure 4A) was observed. However, the heterogeneity was relatively high (I^2^=88.00%, P<0.10, Figure 4A). Sensitivity analysis suggested that Slattery et al.^39^ had significant impact on the result (Figure 4C). After the removal of Slattery et al.^39^, publication bias investigation further identified another outlier, Papageorgioua et al.^38^ (Figure 4D). With the elimination of the two outliers, the heterogeneity greatly decreased (I^2^=25.50%, P=0.225, Figure 4B), and the relation of miR-92a expression level to OS was also significant (Figure 4B). Excluding two studies resulted in eight studies including 1214 Chinese patients remaining in the analysis. Subgroup analysis was performed, as shown in Table 2. It suggested that the homogeneity was achieved within the studies of sample size less than 100, NOS scores greater than or equal to 8, specimen derived from tissues and the patients of non-gastrointestinal cancer. Moreover, all the subgroups revealed the significant association between miR-92a expression level and OS of Chinese cancer patients. Meta regression was also performed, which suggested that none of the subgroup factors could explain the source of heterogeneity significantly.

### The relation of miR-92a expression levels to DFS, RFS and PFS

As shown in Table 3, significant association between miR-92a expression levels and PFS was identified and the relative heterogeneity was not obvious. However, there was no significant relation recognized of miR-92a expression level to RFS of log rank tests, RFS of cox regression by random pooling model chosen for relatively high heterogeneity. DFS of log rank tests and DFS of cox regression were also found to be significantly associated to miR-92a expression level by random pooling model. Furthermore, the fixed pooling model revealed the consistent significance, indicating the stability and reliability of the results.

### Correlations between miR-92a levels and clinicopathological features among various carcinomas

**Figure 5:**
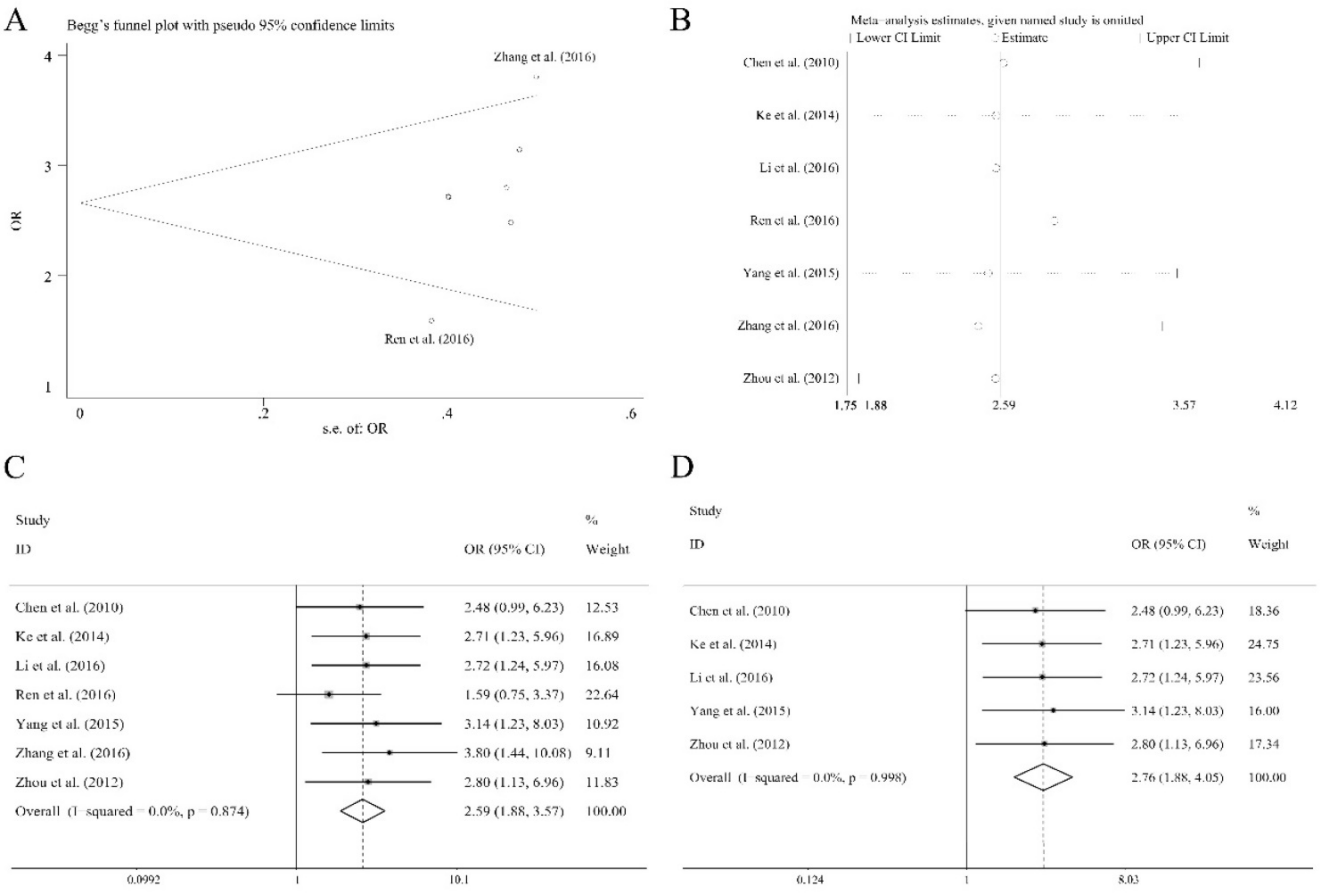
** Association between miR-92a expression level and TNM stages of cancer patients, (A)** publication bias evaluation, **(B)** sensitivity analysis, **(C)** overall pooling result, **(D)** pooling result without the outliers.

There were eleven articles containing 1138 patients of different cancers that investigated several clinical characteristics and the related miR-92a expression level. As shown in Table 4, there were significant relations observed of the expression level of miR-92a to tumor size (≥5 vs <5 cm), lymph node metastasis (present vs. absent), distant metastasis (present vs. absent), TNM stage (III+IV vs. I+II) and differentiation (poor vs. others) by fixed pooling model. Furthermore, the significance was still consistent by random pooling model (Figure S2). However, there were no significance identified in the gender (male vs. female) and age (≥60 vs <60 years) (Table 4). The obvious heterogeneity was only present in the analysis of age (I^2^=73.10%, P=0.024). Sensitivity analysis and evaluation of publication bias were applied to each clinical characteristic analysis. Publication bias evaluation reported obvious results for TNM stages (P=0.072 for Begg test, P=0.054 for Egger test, respectively), and Ren et al.^31^ as well as Zhang et al.^36^ were found to be the source of bias (Figure 5A). In addition, sensitivity analysis revealed no significant findings (Figure 5B). The removal of the two outliers did not alter the significance of the pooling results (the former, OR=2.59, CI: 1.88-3.57, Figure 5C; the latter, OR=2.76, CI: 1.88-4.05, Figure 5D).

## Discussion

The past several decades has witnessed an emerging studies focusing on exploring reliable prognostic biomarkers in order to guide treatment and improve outcomes by informing clinical decision making. The prognostic value of miR-92a has been investigated widely in various types of human cancers. Here, we intended to summarize and assess the findings of published literatures and extract valuable data that can be utilized in clinical decision-making referring to human malignancies.

Survival data for 2573 cancer patients in 20 different studies were comprehensively analyzed. Sixteen studies containing 1944 patients evaluated the effect of miR-92a serving as an indicator for OS using log rank tests. Analyzing the studies comprehensively, the significant association between miR-92a expression levels and OS was identified consequently, which suggested that the over expression of miR-92a might be a risk factor of unfavorable prognosis of cancer patients. However, we also identified significant heterogeneity, which could be introduced from unknown or known sources^46^. Several approaches were implemented to optimize the power of heterogeneity. Subgroup analyses were performed to identify the potential sources of heterogeneity, such as population, sample sizes, NOS scores, specimen, tumor category and so on. As a result, the heterogeneity was greatly reduced within Chinese population, non-hematological cancer, osteosarcoma, non-small cell lung cancer, and homogeneity was achieved within studies of specimen derived from tissues and hepatocellular carcinoma group. Though heterogeneity was not controlled in other groups, such as gastrointestinal cancer, studies of sample sizes greater than or equal to 100 and so on, the random pooling model did not alter the significance in most of the subgroups, indicating the statistical stability of the results. In addition, the sensitivity analysis did not reveal any significant findings, suggesting that there was no study of significant impact on the pooled results. Moreover, the evaluation of publication bias identified two studies that posed obvious deviation, which were Liu et al.^21^ and Papageorgioua et al.^38^ After retrieving the studies, we found the patients recruited in Papageorgioua et al.^38^ were all from Greece, while other studies investigated the Chinese patients. Besides, Papageorgioua et al.^38^ was the only research that focused on the hematological cancer. The two factors mentioned above might contribute to the publication bias. However, we could not highlight the underlying bias from Liu et al.^21^, which might come from the miR-92a detecting methods or the therapeutic variation. The removal of those two studies greatly fuzzed the presence of heterogeneity. Summarily, the elevated miR-92a expression level is associated with unfavorable prognosis of Chinese cancer patients, especially for patients of osteosarcoma, colorectal cancer, non-small cell lung cancer or hepatocellular carcinoma. As for patients from another country or patients of hematological malignance, further relevant researches are required to draw a precise conclusion. Besides, ten articles including 1519 patients studied the independent role of miR-92a playing as the prognostic indicator with cox multivariate regression^47^ by adjusting other factors. Curiously, the significance of overall analysis was not consistent among different pooling model, suggesting the instability of the results. Besides, the presence of heterogeneity was also relatively obvious. Similarly, we applied subgroup analyses, sensitivity analyses, publication bias investigation and meta regression. As a result, the sensitivity analysis and publication bias evaluation identified two outliers, Slattery et al.^39^ and Papageorgioua et al.^38^ which recruited the patients from Utah or California and Greece, respectively, leaving the remaining studies of Chinese cancer patients. The elimination of those two studies^38^, ^39^ significantly optimized the presence of heterogeneity, furthermore, the over expression of miR-92a was significantly related to OS among the Chinese cancer patients. Also, the significance was observed in all the subgroups. Thus, the power of miR-92a expression level serving as an independent prognostic indicator is essential and consistent under those subgroup factors. It should be clarified that with the absence of specific data in text for OS, the HRs and its corresponding confidence intervals of Jiang et al.^26^, Ke et al.^20^ and Lu et al.^25^ were extracted by two independent authors (Lu Tang and Xiangcheng Qing) using the Kaplan-Meier Curves with Engauge Digitizer 9.8 and calculated in the spreadsheet calculator designed by Tierney JF et al.^42^, whose accuracy had been proved by many researches^48^-^50^. The extracted results were always harmonious among the investigators but inconsistent with significance claimed in the original articles. Thus, more precise data extracting methods or improving qualities of the recruited studies was required to avoid the bias. PFS, DFS, RFS were also taken into account. MiR-92a expression level was found to be significantly associated with PFS and DFS of statistics extracted from both the log rank tests and cox regression analysis. However, the significance was not observed in RFS. It was noticed that two studies^29^, ^37^ recruited for RFS analysis had included Swedish patients of breast cancer and Chinese patients of hepatocellular carcinoma, respectively. It suggested that the variable racial genetic background might pose an impact on the prognostic efficacy of miR-92a levels, besides the tumor type. Due to insufficient enrolled studies, subgroup, sensitivity analyses and publication bias evaluation were not performed.

As for the clinical features, eleven articles including 1021 Chinese patients and 117 Swedish patients have evaluated the relation of miR-92a to the specific clinical features. The over expression of miR-92a was found to be significantly related to larger tumor size, greater potential of lymph node metastasis and tumor distant metastasis, more advanced TNM stages and poorer differentiation degree. These results were consistent to the current findings. MiR-92a has been found to act as an oncogenic-miRNA and contribute to the cancer cells proliferation^51^-^53^ and invasion activity^54^. In addition, sensitivity analyses did not recognize any studies of significant impact on those results. However, publication bias identified two researches, Ren et al.^31^ and Zhang et al.^36^, for TNM stages analysis. The removal of those studies did not alter the significance of the result. Since the number of enrolled studies for the analysis of certain clinical features was still inadequate, more relevant researches were demanded to enrich the results. Moreover, clinical features of a specific cancer should be quantified and normalized based on a certain standard, such as the cut-off values, the feature categories and so on, so as to enlarge the enrolled cases and characteristics for the meta-analysis. According to our findings, it is safe to demonstrate that patients of elevated miR-92a expression level are likely to develop the cancer of more malignant behavior.

Although Liu et al. 55 and Zhang et al.^56^ have made a meta-analysis studying the relationship between miR-17-92 cluster (miR-17, miR-18a, miR-19a/b, miR-20a, and miR-92a) and human cancers, both of them only focus on the overall effects of all six mi-RNAs on cancer, instead of further analyzing the prognostic value of each mi-RNA based on the detailed information, such as different specimens, sample sizes, cancer categories, etc. Also, the correlation between each miRNA expression and clinicopathological characteristics of cancer patients was not considered in both of the studies. Thus, the exact role of miR-92a on the clinical prognosis of patients in various human cancers still needs further recognition.

Apart from the 20 articles we have included, there were 2 articles that also contained the prognostic data (Cun et al.^57^ and Chen et al.^58^). Interestingly, the survival data in Cun et al.^57^ was from Kaplan-Meier Plotter Database (KMPD), an online survival analysis tool whose data sources are from gene expression omnibus (GEO), the cancer genome Atlas TCGA), European genome-phenome archive (EGA), and PubMed, and data for Chen et al.^58^ was extracted from TCGA Colon and Rectal Cancer (COADREAD) data. However, we found that those data should not be included in the meta-analysis for the following results: (1) we could not ensure whether there were no overlaps among the previous data and the data form the online database; (2) For lack of the detail of those online data, for example, the patient selection and comparability et al., we could not perform data evaluation which is essential for meta-analysis. Even though data from those articles should not be included for statistical analysis, the results drawn from Cun et al.^57^ also supported our conclusion. More specifically, Kaplan-Meier survival analysis revealed significantly reduced overall survival in breast cancer patients with high miR-92a expression. Moreover, Chen et al.^58^ found that though the prognostic difference was not significant, increasing trends of miR-92 level was identified in lymph node involvement, metastasis and advanced pathology of colorectal cancer.

To our knowledge, this meta-analysis was the most comprehensive and systematic meta-analysis to explore the association between the expression level of miR-92a and the prognosis of cancer patients in depth. Rigorous and strategic approaches, such as subgroup analysis, sensitivity analysis, publication bias evaluation, meta regression, etc. have been applied to identify possible bias and eliminate heterogeneity to the greatest extent. However, only the articles in English or Chinese were under inspection, which might lead to deviations in some extents for lack of other races. The number of recruited studies for PFS, RFS, DFS and clinical features analyses were relatively insufficient, which requires more associated researches to be performed and enrolled, so as to improve the stability and reliability of the findings.

## Conclusions

Overexpression of miR-92a is associated with unfavorable prognosis of Chinese cancer patients, especially for patients of osteosarcoma, colorectal cancer, non-small cell lung cancer or hepatocellular carcinoma. As for patients from other countries or patients of hematological malignance, further relevant researches are required to draw a precise conclusion. In addition, patients of elevated miR-92a expression level are likely to develop the cancers of more malignant behavior, such as larger tumor size, greater potential of lymph node metastasis and tumor distant metastasis, more advanced TNM stages and poorer differentiation degree.

## Supplementary Material

Supplementary figures.

Table S1.

## Figures and Tables

**Table 1:** Association between miR-92a expression levels and overall survivals

	No. of studies	No. of patients	Pooled HR(95%CI)			Meta regression			Heterogeneity	
			Fixed	Random		p-value^#^	p-value^*^		I^2^	p-value
**Overall**	16	1944	2.18(1.87,2.53)	2.14(1.57,2.92)					72.40%	0.000
**Population**						0.184	0.174			
Chinese	15	1856	2.35(2.02,2.74)	2.41(1.91,3.04)					47.80%	0.020
Greek	1	88	0.28(0.13,0.61)	0.28(0.13,0.61)					-	-
**Sample Size**						0.723	0.932			
<100	8	592	1.90(1.45,2.49)	1.83(1.05,3.20)					76.10%	0.000
≥100	8	1352	2.32(1.93,2.77)	2.43(1.68,3.54)					70.40%	0.001
**NOS Scores**						0.691	0.547			
<8	8	1023	1.88(1.54,2.29)	1.76(1.09,2.84)					79.90%	0.000
≥8	8	921	2.67(2.12,3.36)	2.64(1.83,3.83)					51.10%	0.046
**Specimen**						0.154	0.158			
tissues	12	1161	2.43(1.99,2.96)	2.43(1.99,2.96)					0.00%	0.713
blood	4	783	1.88(1.49,2.36)	1.81(0.68,4.82)					93.10%	0.000
**Tumor Category 1**						0.574	-			
Gastrointestinal cancer	8	1197	2.24(1.83,2.75)	2.50(1.65,3.79)					69.50%	0.002
Non-gastrointestinal cancer	8	747	2.10(1.69,2.63)	1.81(1.09,3.00)					77.60%	0.000
**Tumor Category 2**						-	0.442			
Hematological cancer	1	88	0.28(0.13,0.61)	0.28(0.13,0.61)					-	-
Non-hematological cancer	15	1856	2.35(2.02,2.74)	2.41(1.91,3.04)					47.80%	0.020
**Tumor**						-	-			
esophageal squamous cell carcinoma	2	170	1.82(1.07,3.10)	1.78(0.72,4.41)					65.30%	0.090
osteosarcoma	2	131	2.49(1.38,4.48)	2.54(1.26,5.12)					28.40%	0.237
colorectal cancer	4	514	3.45(2.28,5.24)	3.67(1.87,7.20)					56.40%	0.076
non-small cell lung cancer	2	246	2.65(1.90,3.70)	2.33(1.21,4.51)					40.80%	0.194
chronic lymphocytic leukemia	1	88	0.28(0.13,0.61)	0.28(0.13,0.61)					-	-
gastric cancer	2	513	1.99(1.53,2.58)	2.03(0.98,4.18)					86.90%	0.006
hepatocellular carcinoma	2	196	2.40(1.56,3.72)	2.40(1.56,3.72)					0.00%	0.866
nasopharyngeal carcinoma	1	86	2.17(1.00,4.72)	2,17(1.00,4.72)					-	-

Abbreviations: 95%CI, 95% confidence interval; Fixed, fixed pooling model; Random, random pooling model; HR, hazard ratio; NOS, Newcastle-Ottawa scale scores; #, the covariates for meta-regression are population, sample size, NOS scores, specimen, tumor category 1; *, the covariates for meta-regression are population, sample size, NOS scores, specimen, tumor category 2.

**Table 2:** Meta-analysis of miR-92a as an independent prognostic indicator for Chinese patients of various carcinomas

	No. of studies	No. of patients	Pooled HR(95%CI)			Meta regression		Heterogeneity	
			Fixed	Random		p-value		I^2^	p-value
**Overall**	8	1214	2.02(1.66,2.46)	2.10(1.65,2.67)				25.50%	0.225
**Sample Size**						0.097			
<100	3	233	2.13(1.46,3.11)	2.13(1.46,3.11)				0.00%	0.945
≥100	5	981	1.99(1.58,2.50)	2.21(1.50,3.25)				56.50%	0.057
**NOS Scores**						0.091			
<8	3	619	1.75(1.28,2.38)	2.03(1.12,3.67)				66.20%	0.052
≥8	5	595	2.23(1.73,2.89)	2.23(1.73,2.89)				0.00%	0.728
**Specimen**						0.079			
tissues	5	519	2.35(1.73,3.18)	2.35(1.73,3.18)				0.00%	0.850
blood	3	695	1.81(1.40,2.35)	2.00(1.18,3.39)				68.90%	0.040
**Tumor Category**						0.290			
Gastrointestinal cancer	5	826	1.94(1.47,2.56)	2.29(1.44,3.64)				56.20%	0.058
Non-gastrointestinal cancer	3	388	2.11(1.60,2.80)	2.11(1.60,2.80)				0.00%	0.959

Abbreviations: 95%CI, 95% confidence interval; Fixed, Fixed pooling model; Random, Random pooling model; HR, hazard ratio; NOS: Newcastle-Ottawa scale scores

**Table 3:** Association between miR-92a expression levels and other prognostic indicators

	No. of studies	No. of patients	Pooled HR(95%CI)			Heterogeneity	
			Fixed	Random		I^2^	p-value
**PFS**	2	295	3.17(1.79,5.63)	3.28(1.68,6.43)		21.00%	0.261
**RFS**							
univariate	2	223	1.42(0.91,2.21)	0.92(0.13,6.43)		93.30%	0.000
multivariate	2	223	1.47(0.80,2.69)	1.20(0.13,11.31)		92.50%	0.000
**DFS**							
univariate	3	615	1.89(1.54,2.32)	1.93(1.25,2.97)		72.60%	0.026
multivariate	3	615	1.76(1.41,2.19)	1.85(1.26,2.72)		61.00%	0.077

Abbreviations: 95%CI, 95% confidence interval; Fixed, Fixed pooling model; Random, Random pooling model; HR, hazard ratio; NOS, Newcastle-Ottawa scale scores

**Table 4:** Overall analysis of miR-92a expression association with clinicopathological characteristics.

Clinicopathological parameters	No. of studies	No. of patients	Pooled OR (95%CI)			Heterogeneity	
			Fixed	Random		I^2^	p-value
Gender (male vs. female)	10	1063	0.87(0.64,1.17)	0.87(0.63,1.20)		10.40%	0.347
Age (≥60 vs <60 years)	3	395	1.33(0.83,2.13)	1.36(0.52,3.55)		73.10%	0.024
Tumor Size (≥5 vs <5 cm)	3	293	2.13(1.31,3.45)	2.13(1.31,3.45)		0.00%	0.857
Lymph node metastasis (present vs. absent)	5	581	1.87(1.31,2.69)	1.91(1.15,3.17)		47.30%	0.108
Distant metastasis (present vs. absent)	7	745	2.99(1.77,5.03)	2.91(1.72,4.92)		0.00%	0.823
TNM stage (III+IV vs. I+II)	7	826	2.59(1.88,3.57)	2.58(1.87,3.56)		0.00%	0.874
Differentiation (poor vs. others)	5	455	1.75(1.07,2.85)	1.75(1.07,2.85)		0.00%	0.999

Abbreviations: 95%CI, 95% confidence interval; Fixed, Fixed model; OR, odds ratio; Random, Random model.

## Abbreviations

CNKI: the China National Knowledge Infrastructure
DFS: disease-free survival
HR: hazard ratio
HRs: pooled hazard ratios
miRNAs: microRNAs
NOS: the Newcastle-Ottawa Scale
OS: overall survival
PFS: progression-free survival
qRT-PCR: Real-time Polymerase Chain Reaction
RFS: recurrence-free survival
WOS: Web of Science
95%Cis: 95% confidence intervals
