# Supplementary material for: Investigation of MiR-92a as a Prognostic Indicator in Cancer Patients: a Meta-Analysis
Source: J Cancer. 2019 Jul 23;10(18):4430–41. doi: 10.7150/jca.30313 (PMC6691717; doi:10.7150/jca.30313)
Supplement: Supplementary file 1 — Supplementary figures. [file jcav10p4430s1.pdf]

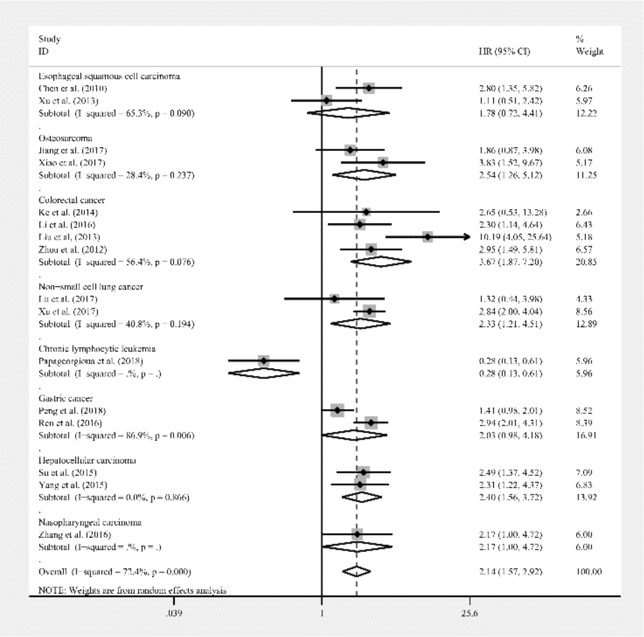

Figure S1 Association between miR-92a expression levels and overall survival in various types of carcinomas

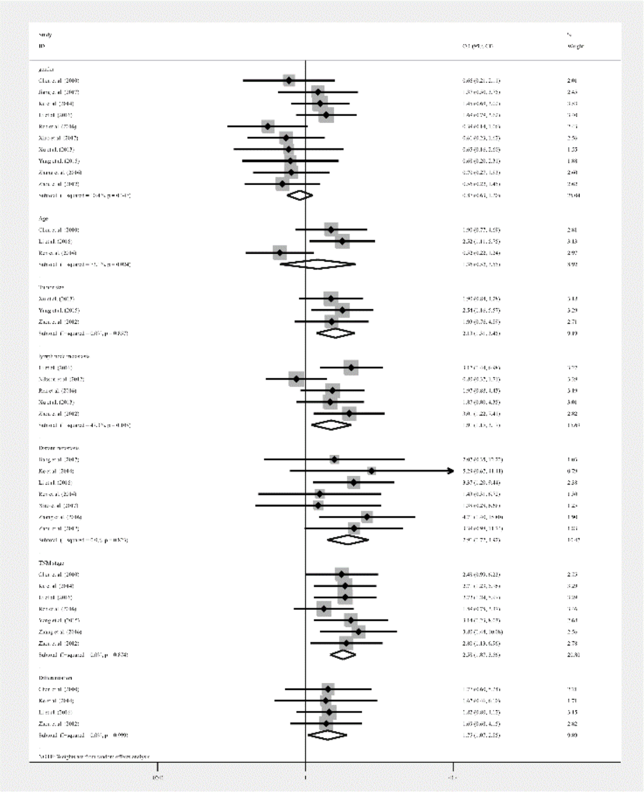

Figure S2 Relation of miR-92a to several clinical characteristics of cancer patients
